# Detecting mir-155-3p through a Molecular Beacon Bead-Based Assay

**DOI:** 10.3390/molecules29133182

**Published:** 2024-07-03

**Authors:** David Moreira, Daniela Alexandre, André Miranda, Pedro Lourenço, Pedro V. Baptista, Cândida Tomaz, Yi Lu, Carla Cruz

**Affiliations:** 1 CICS-UBI—Health Sciences Research Centre, University of Beira Interior, 6201-506 Covilhã, Portugal; david.moreira@ubi.pt (D.M.); danilex.ct3@gmail.com (D.A.); andre.miranda@ubi.pt (A.M.); pedro.afonso.amaro.lourenco@ubi.pt (P.L.); 2 UCIBIO, Department of Life Sciences, Faculdade de Ciências e Tecnologia, Universidade NOVA de Lisboa, 2829-516 Caparica, Portugal; pmvb@fct.unl.pt; 3 i4HB, Associate Laboratory, Institute for Health and Bioeconomy, FCT-NOVA, 2829-516 Caparica, Portugal; 4 Departamento de Química, Universidade da Beira Interior, Rua Marquês de Ávila e Bolama, 6201-001 Covilhã, Portugal; ctomaz@ubi.pt; 5 Department of Chemistry, The University of Texas at Austin, Austin, TX 78712, USA; yi.lu@utexas.edu

**Keywords:** lung cancer, molecular beacon, microfluidics, mir-155-3p

## Abstract

Lung cancer (LC) is recognized as one of the most prevalent and lethal cancers worldwide, underscoring an urgent need for innovative diagnostic and therapeutic approaches. MicroRNAs (miRNAs) have emerged as promising biomarkers for several diseases and their progression, such as LC. However, traditional methods for detecting and quantifying miRNAs, such as PCR, are time-consuming and expensive. Herein, we used a molecular beacon (MB) bead-based assay immobilized in a microfluidic device to detect miR-155-3p, which is frequently overexpressed in LC. The assay relies on the fluorescence enhancement of the MB upon binding to the target miRNA via Watson and Crick complementarity, resulting in a conformational change from a stem–loop to a linear structure, thereby bringing apart the fluorophores at each end. This assay was performed on a microfluidic platform enabling rapid and straightforward target detection. We successfully detected miR-155-3p in a saline solution, obtaining a limit of detection (LOD) of 42 nM. Furthermore, we evaluated the method’s performance in more complex biological samples, including A549 cells’ total RNA and peripheral blood mononuclear cells (PBMCs) spiked with the target miRNA. We achieved satisfactory recovery rates, especially in A549 cells’ total RNA.

## 1. Introduction

Cancer is recognized by the World Health Organization as one of the principal health challenges of the 21st century [1,2]. Lung Cancer (LC) is one of the most common and deadly cancers worldwide [3], and it may be divided into non-small-cell lung cancer (NSCLC) and small-cell lung cancer (SCLC) [4]. The former is the most prevalent and like other cancers, it has a multifactorial origin (genetic, environmental) [5]. Current challenges regarding NSCLC include the development of more effective therapies with fewer side effects [6] and diagnosis systems that allow for the early detection of LC [7]. 

Focusing on improving the effectiveness of detecting LC at early stages, microRNAs (miRNAs) have emerged as potential biomarkers since they may be up- or down-regulated in LC and may present different expressions in distinct stages of the disease [8,9]. These miRNAs are single-stranded non-coding RNAs with a length of between 21 and 23 nucleotides that are important in regulating gene expression by silencing mRNA [10]. Regarding their importance in regulatory processes, they are involved in mechanisms that lead to NSCLC development, such as cell proliferation, metastasis, and apoptosis resistance, acting either as tumor suppressors or oncogenic miRNAs [11,12]. Thus, several investigations are underway to define miRNA profiles for various diseases and devise effective methods for their detection and quantification. 

Regarding the miRNAs dysregulated in LC, a systematic review of the literature was performed by Yu et al. to identify potential biomarkers to help diagnose LC in Western populations [13]. In this review, 109 miRNAs were found to be dysregulated, with mir-21 showing the most consistent overexpression pattern. Other notable miRNAs with altered expression, including miR-155, miR-182, miR-203, miR-205, and miR-25. Among these, miR-155-3p has been specifically highlighted. Our group has already shown that miR-155-3p is up-regulated in peripheral blood mononuclear cells (PBMCs) extracted from NSCLC patients [14], suggesting its potential as a promising biomarker for LC. This dysregulation has also been reported in other types of biological samples, although it is important to note that the dysregulation observed in PMBCs may differ from that observed in plasma or tissues [15]. Furthermore, miR-155-3p is noted for its role in tumorigenesis, proliferation, invasion, and angiogenesis [16], and its knockdown leads to the inhibition of cell proliferation, migration, and invasion in NSCLC cells [17], suggesting its potential in LC treatment.

In the quest for more effective methods to detect and quantify miRNAs, molecular beacons (MBs) have risen as promising tools due to their advantageous characteristics in detecting nucleic acids [18]. Their working principle is based on their stem–loop structure that forces the fluorophores on their extremities to be very near to each other via stem sequence complementarity. This spatial proximity results in fluorescence quenching. Then, the detection of the target nucleic acid relies on the complementarity of the target, with the bases forming the loop region of the MB. This complementarity will result in a strong interaction of the target with the MB, leading to the split of the stem region. This separation of the stem sequences, resulting from the hybridization of the target with the MB, will bring the fluorophores of each strand apart, resulting in a fluorescence signal [19,20]. Several approaches have been developed to take advantage of MB features through incorporation into microfluidic devices (Lab-on-a-chip) that allow for easy in situ detection of a molecule of interest in a fast way. 

Here, we present a microfluidic device fabricated on polydimethylsiloxane (PDMS) designed to have two-channel widths, allowing us to trap the beads inside the channel. This device allows for the detection and quantification of miR-155-3p using an MB bead-based assay immobilized in a microfluidic device, resulting in a fast and straightforward approach to detecting this miRNA.

## 2. Results and Discussion

**Figure 1:**
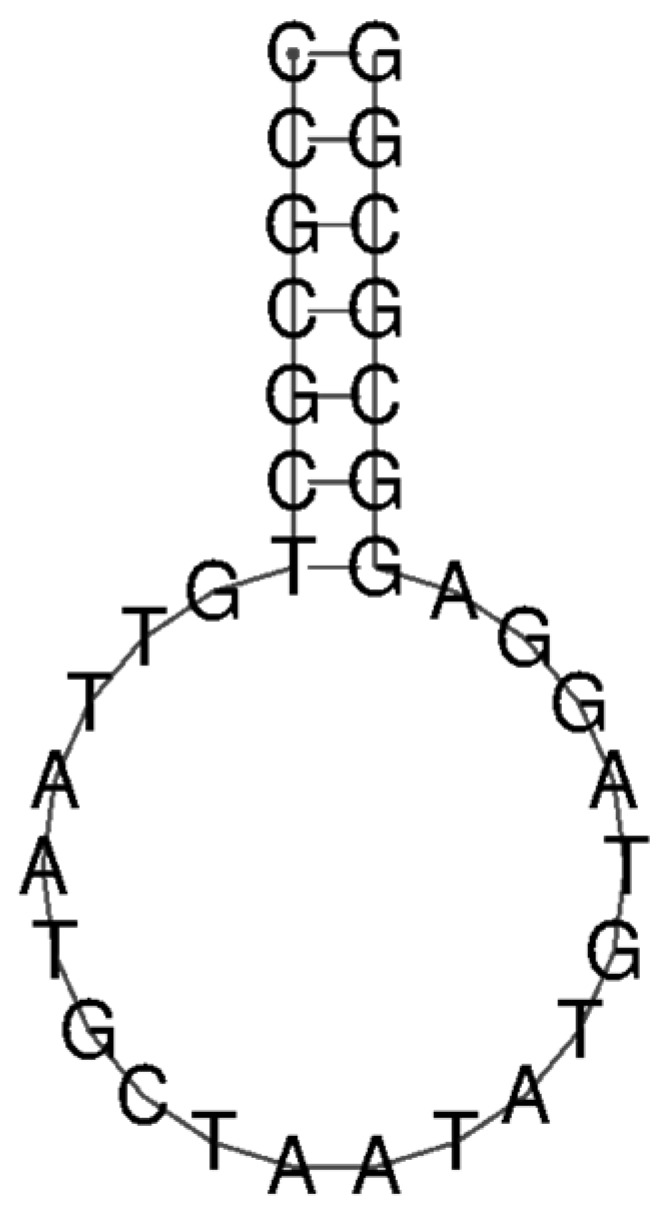
Prediction of MB structure through RNAfold software (Version 2.6.4) [21].

According to previous findings that mir-155-3p is overexpressed in NSCLC patients [14], we developed an MB intended to adopt a stem–loop structure, with the loop sequence specifically designed to be complementary to that of miR-155-3p. The structural prediction of the MB was conducted using RNAfold by Vienna RNA [21], as shown in Figure 1. Further biophysical characterization of the MB has already been reported by Alexandre et al. [22]. 

As expected, the MB adopts a stem–loop structure, allowing the fluorophores to be in spatial proximity, resulting in fluorescence quenching. A biotin modification was introduced at the 5′ end to allow for the functionalization of the streptavidin beads. The efficiency of the functionalization, based on the quantification of the non-bound MB obtained from the centrifugation’s supernatant, was 60% (total of 12 μM immobilized in beads).

**Figure 2:**
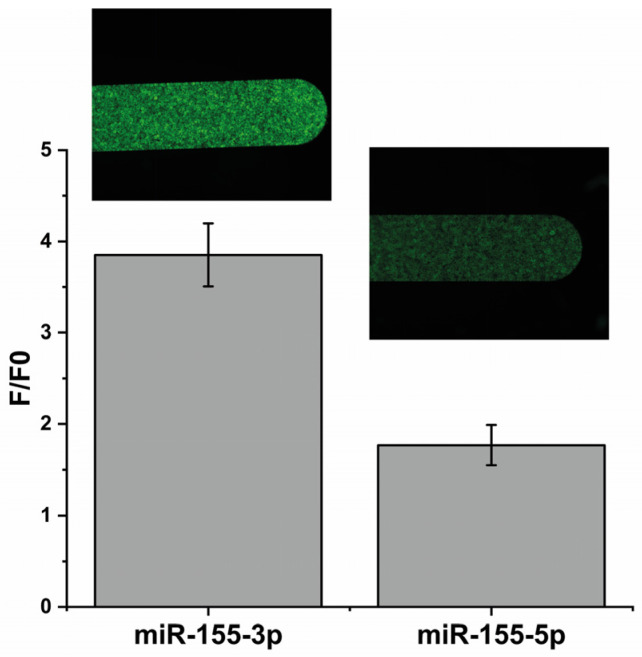
A comparison between the fluorescence intensities obtained using 12 μM of the target (mir-155-3p) and 12 μM of a different miRNA (miR-155-5p) to check MB specificity.

To understand if the MB could detect the miR-155-3p in a specific way, we first performed an assay where a solution containing a considerable concentration (12 μM; 1 molar equivalent of immobilized MB) of mir-155-3p was passed through the channel with the MB-functionalized beads. Simultaneously, a solution with an equivalent concentration of a distinct miRNA (miR-155-5p) was introduced in another channel. As expected, in Figure 2, the increase in the fluorescence resulting from the interaction with the mir-155-3p is about 2.2 times higher than with 155-5p, showing that the MB detects mir-155-3p in a specific way.

**Figure 3:**
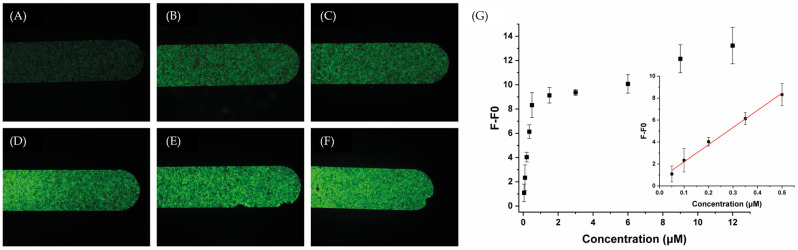
Representative fluorescence images of the microfluidics channels packed with the MB after injecting different concentrations of miR-155-3p: (**A**) 0 nM, (**B**) 50 nM, (**C**) 100 nM, (**D**) 200 nM, (**E**) 350 nM, and (**F**) 500 nM, and (**G**) a calibration plot of fluorescence intensity versus miR-155-3p concentration. The inset exhibits the linear relationship between the fluorescence response and target concentration.

To investigate the system’s sensitivity, fluorescence intensity was recorded upon the addition of increasing concentration of miR-155-3p until reaching a plateau phase. The titration began at a concentration of 0.05 μM of the target, demonstrating a linear response up to 0.50 μM. From 0.50 μM to 12 μM, the system reached a plateau phase, as depicted in Figure 3. Based on this observation, we conclude that within the linear range (0.05–0.50 μM), fluorescence exhibits a concentration-dependent linear behavior. The corresponding regression equation was found to be F − F0 = 15.645C + 0.629, with a correlation coefficient R^2^ of 0.994, where C is the concentration (in μM) of the miR-155-3p, F is the fluorescence intensity at concentration C, and F0 is the fluorescence intensity of the blank (1× PBS without any miR-155-3p). The detection limit (LOD) is 42 nM (estimated from 3σ/k, where σ is the standard deviation of blanks and k is the slope of the standard curve).

Despite the obtained LOD being higher than that reported in other works (Table 1) that intended to detect miR-155-3p, which achieved LODs in the fM order [23,24], this result is still promising regarding the method utilized. When compared to similar techniques, such as those employing an MB immobilized on beads or similar surfaces, our approach demonstrates an LOD in the same order [25,26]. However, as our method is based on a user-friendly microchip, it offers the advantage of easy quantification without the need for highly sophisticated materials. This represents a step toward the in situ detection and quantification of miR-155-3p. This method also has the potential for easy adaptation to other target miRNAs and, in the future, could facilitate the detection of multiple targets by creating distinct MBs for each target with different fluorophores.

**Figure 4:**
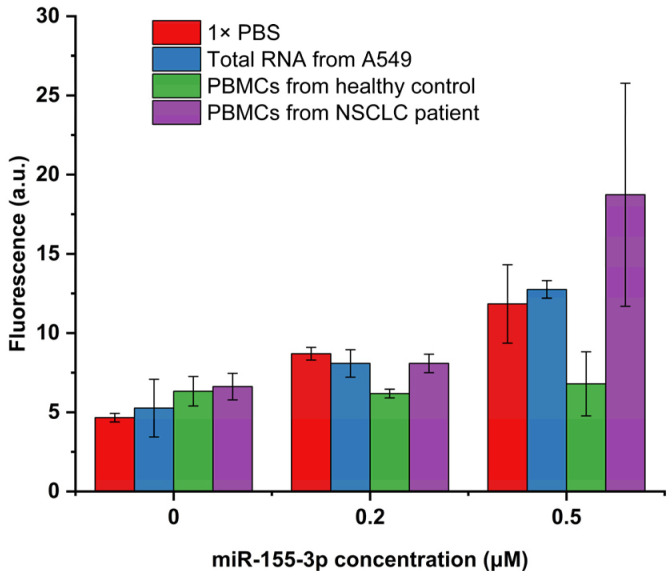
Fluorescence intensities obtained on biological samples spiked with 0, 0.2, and 0.5 μM of miR-155-3p (*n* = 3).

To further verify the reproducibility of the proposed detection system for the analysis of complex biological samples, a detection test was conducted on total RNA extracted from A549 cells spiked with different concentrations of miR-155-3p and on the PBMCs’ lysate (from NSCLC patients and healthy controls) also spiked with different concentrations of the target. Regarding the samples of PBMCs and total RNA from A549 cells (Figure 4), without spiking, we can observe a small increase in basal fluorescence, and the standard deviation of this fluorescence increases. This variation is less evident in the total RNA extracted from A549 cells, which is a good indicator of the MB’s selectivity for our target RNA. It is not a surprise that on PBMCs, the fluorescence increase is higher since this sample is much more complex than the 1× PBS used as a blank. However, this increase is not considerable and can result from non-specific interactions between the numerous components of the PBMCs’ lysate.

When looking at the spiked samples, the behavior of the detection system varies significantly depending on the type of spiked sample used. Indeed, the sample type that allows for better system performance is the total RNA from A549 cells. This result is not unexpected, considering that this sample is more complex than PBS but considerably less complex than the PBMCs’ lysate. Regarding the recovery results of the spiked A549 cells’ total RNA (Table 2), the detection system showed good performance, enabling the detection of the lowest concentration used (0.2 μM) with a recovery of 100.42% and the detection of the highest concentration used (0.5 μM) with a recovery of 108.16%. However, in the case of PBMCs, the performance of the detection system was much less satisfactory. The PBMC samples extracted from the healthy controls (Table 2) yielded recovery percentages of only 31.14% when spiked with 0.2 μM and 21.39% when spiked with 0.5 μM. As previously discussed, the complexity of the PBMC samples surpasses that of total RNA from A549 cells and PBS, potentially resulting in an increased LOD. This complexity may lead to non-specific interactions between the MB and other components, such as proteins, which elevate the basal fluorescence and hinder the interaction between the MB and our miRNA. This behavior was already reported in other MB-based assays that reported a good performance on MCF7 cells but lower fluorescence recovery on serum samples [31]. This change in the fluorescence detected on the serum samples was also reported when using qRT-PCR performed in the same study, indicating that variations in the fluorescence recovered will always depend on the complexity of our sample. 

However, when testing PBMCs extracted from NSCLC patients (Table 1), the recovery percentages obtained are much more accurate than those from healthy patients. Specifically, recovery percentages of 100.42% were obtained when spiked with 0.2 μM and 195.22% when spiked with 0.5 μM. This result is not expected since the interference of the PMBCs’ complexity on the detection system should theoretically remain the same irrespective of the health condition of the PBMCs’ donor. 

These performance differences in detecting miR-155-3p in PBMCs may also be explained by its overexpression in NSCLC patients, which can be up to 3.19 times higher [14]. Consequently, PBMCs extracted from NSCLC patients may inherently contain a higher target miRNA concentration than spiked samples. This higher target concentration may also overcome some steric impediments arising from the accumulation of proteins near the MB due to non-specific interactions.

Overall, the proposed detection system has advantages and disadvantages that must be considered in developing future detection systems. Starting from the advantages, the microfluidic system offers a straightforward assay with a short procedure time (several minutes vs. several hours on standard assays such as PCR). It requires minimal manipulation and is cost-effective in terms of both reagents and the needed material for fluorescence detection. Moreover, it demonstrates the capability to detect and quantify miR-155-3p with high selectivity and specificity in simple samples or total RNA extracted from cells. Finally, it is remarkable that this system can be used virtually in the future to detect any miRNA simply by constructing a new streptavidin MB that is complementary to the desired target miRNA. Moreover, this approach can even be used to detect several miRNAs simultaneously by multiplexing them using a mix of MBs to detect several biomarker miRNAs. To achieve this, each MB should be functionalized with a different fluorophore to detect different miRNAs at different wavelengths. As an alternative to surpass possible interferences by using several fluorophores and quenchers, it is also possible to connect several channels in series. After that, each channel should be functionalized with an MB for a specific miRNA using the same fluorophore. The sample will then be injected through all of the connected channels, and if an increase in fluorescence is detected in a channel, a specific miRNA will be detected. Some approaches are also described intending to use similar methods to profile miRNAs [32,33]. 

On the other hand, disadvantages include the performance of more complex samples like PMBCs and the relatively high LOD compared with other available systems. However, these limitations could be surpassed in future similar approaches by integrating a signal amplification mechanism into the system or implementing PMBC processing techniques. These modifications would simplify the sample, enabling more accurate detections and quantifications.

## 3. Materials and Methods

### 3.1. Oligonucleotides and Ligands

All chemicals, reagents, and solvents were purchased from commercial sources (Sigma Aldrich, St. Louis, MO, USA; Fisher Scientific, Hampton, NH, USA; Merck, Darmstadt, Germany). Oligonucleotides (miR-155-3p, miR-155-5p, and MB) were obtained from Eurogentec with HPLC purification and their sequences are depicted in Table 3. Streptavidin Sepharose beads were acquired from GE Healthcare (Chicago, IL, USA). PDMS microfluidic devices were fabricated as previously described [26,34]. 

### 3.2. Bead Conjugation with MB

The MB was diluted at a concentration of 20 µM on a 1× phosphate-buffered saline (PBS) solution of pH 7.1 and annealed by heating it at 95 °C for 5 min, with a subsequent stem quench on ice for 10 min. Streptavidin Sepharose beads were firstly washed with 300 µL of absolute ethanol and centrifugated for 1 min at 7500 rpm. Ethanol was removed and the beads were rinsed on 500 µL of PBS and centrifugated again for 1 min at 7500 rpm. 

PBS was removed and the beads were incubated on the previously annealed MB solution (total of 200 µL) for 2 h. The non-bound MB was removed through centrifugation for 1 min at 7500 rpm. The non-bound fraction was quantified by fluorescence spectroscopy on a Horiba FluoroMax4 fluorometer (Horiba, Kyoto, Japan) with the following parameters: excitation wavelength—495 nm, with a slit of 1 nm; emission wavelength—520 nm, with a slit of 1 nm. This quantification was used to calculate the efficiency of the conjugation.

### 3.3. Cell Culture and Total RNA Extraction

A549 cells were maintained in Ham’s F12 medium (Gibco, Waltham, MA, USA) supplemented with 10% fetal bovine serum (FBS) (Gibco, Waltham, MA, USA) and 1% streptomycin–penicillin (SP) (Gibco, Waltham, MA, USA) antibiotic in a humidified atmosphere at 37 °C and 5% CO_2_. When 80–90% confluency was reached, the cells were harvested and collected. Small RNA molecules were extracted and purified according to the instructions of the miRNeasy Mini Kit from Qiagen (Hilden, Germany). Quantification of the extracted total RNA was achieved using a Nano Photometer from IMPLEN (Munich, Germany). 

### 3.4. Peripheral Blood Mononuclear Cells (PBMCs)

Fresh whole-blood samples were obtained and collected in ethylenediaminetetraacetic acid (EDTA)-coated tubes from Sigma-Aldrich. The procedure for isolating PBMCs involved initial centrifugation of the blood samples in a Beckman Coulter Allegra X-22R at 3000 rpm for 15 min at room temperature. Following centrifugation, the plasma fraction was removed, and an equivalent volume of PBS was added to the tube, with subsequent gentle mixing by inverting the tube. The resulting mixture was carefully transferred into a 50 mL polypropylene tube containing an equivalent volume of Pancoll separating solution. This composite was then centrifuged at 2200 rpm for 30 min at room temperature. The PBMCs, forming a distinct ring in the interphase between Pancoll and the blood fraction, were aspirated using a plastic Pasteur pipette and transferred to a new 50 mL polypropylene tube. Subsequently, 1× PBS was added to the tube, and the mixture was centrifuged at 1800 rpm for 10 min at 4 °C. After discarding the supernatant, the pellet was resuspended in 10 mL of 1× PBS and subjected to centrifugation at 1500 rpm for 10 min at 4 °C. Following removal of the supernatant, the pellet was resuspended in 10 mL of pre-warmed red blood cell lysis solution and gently stirred at 37 °C for 10 min. The sample was then centrifuged at 1500 rpm for 10 min at room temperature. The resulting pellet underwent two washes with 1× PBS through centrifugation at 1500 rpm for 10 min at room temperature. Finally, the PBMCs were resuspended in 1 mL of 1× PBS for cell counting and subsequently stored at −80 °C until further use. Before their use, the PBMCs were lysed at a lysis buffer volume ratio of 1:1 and centrifuged for 1 min at 10,000 rpm, and the supernatant was collected. The protocol to collect blood was approved by the Ethics Committee of University Hospital Center Cova da Beira (35/2019).

### 3.5. Microfluidics Experiments

**Figure 5:**
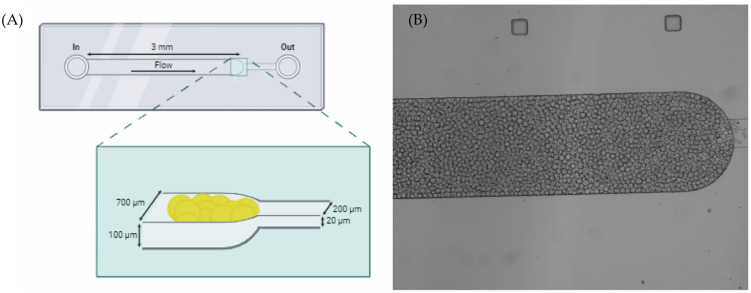
(**A**) Schematic illustrations of the microfluidic device’s design on PDMS composed of two connected microchannels with different widths allowing for the trapping of the beads [26,34]. (**B**) The top view of the microchannels under a widefield microscope.

Microfluidics experiments were based on previously published articles [26,34], as represented in Figure 5. Briefly, the solutions of interest were inserted on a pipette tip and inserted on the inlet. Then, these were forced to enter the microchannel using a syringe pump exerting a negative pressure at the outlet (NE-1002X, New Era Pump System Inc., Farmingdale, NY, USA). First, the functionalized beads were suspended in a solution of 20% (*w*/*w*) polyethylene glycol 8000 (PEG) at a volume ratio of 1:9 (beads/PEG solution). This mix was packed on the microchannel using a 7 μL/min flow rate followed by 1× PBS washing at the same flow rate. Then, the samples (synthetic miRNA solutions, total RNA of A549 cells, or PBMCs) were driven through the microchannel at a flow rate of 1 μL/min to allow for interaction between the target and the MB on the beads. To remove non-specific bound molecules, a final wash with 1× PBS was performed at a flow rate of 5 μL/min. Fluorescence images were then immediately collected on a widefield Axio Observer Z1 inverted microscope with a 5×/0.16 M27 objective using 492 nm as the excitation wavelength (BP 450/90 filter) and collecting the emission at 518 nm (BP 500/50 filter). The images were processed with Zeiss Zen Software 3.7 and ImageJ 1.54. Fluorescence emission values were obtained by averaging the entire end section of the microchannels.

## 4. Conclusions

The microfluidic device based on MB beads enabled the detection of miR-155-3p by increasing fluorescence. The incorporation of the beads into a microfluidic device offers a fast, easy, and cost-effective detection method, requiring minimal handling and materials. These features enable the linear detection of miR-155-3p in a concentration range from 0.05 μM to 0.50 μM with an LOD of 42 nM. The detection of miR-155-3p was also successfully achieved in spiked biological samples.

The microfluidic device’s design offers several advantages. It is highly sensitive, providing quantification of miR-155-3p levels, which is crucial for early detection. Its portability and ease of use make it suitable for point-of-care testing. Additionally, low reagent consumption and minimal sample preparation are required to reduce the costs and complexity of the detection process.

## Figures and Tables

**Table 1:** A comparison of the developed microfluidic device with previously reported methods for miRNA detection.

Detection System	LOD	Linear Range	Target	REF
Fluorescent MB	0.7 pM	5 pM to 40 nM	miRNA-21	[18]
DNAzyme Nanogel	2 pM	2 pM to 500 pM	miRNA-21	[27]
Electrochemiluminescent MB	10 fM	10 fM to 10 nM	let-7d	[28]
Colorimetric gold nanoparticles	10 nM	10 nM to 100 µM	miRNA-155	[25]
PNA microarray	43 pM	0.1 nM to 10 nM	miRNA-122	[29]
SPR sensor based on DNA super-sandwich	9 pM	10 pM to 1 µM	miRNA-21	[30]
Microfluidic molecular beacon bead-based assay	42 nM	0.05 μM to 0.50 μM	miR-155-3p	Current study

**Table 2:** Recovery test of miR-155-3p spiked in PMBCs and serum.

Sample	miR-155-3p Added (μM)	% miR-155-3p
RNA from A549 cells	0.2	100.42
	0.5	108.16
PMBCs from healthy controls	0.2	31.14
	0.5	21.39
PMBCs from NSCLC patients	0.2	100.42
	0.5	195.22

**Table 3:** A list of all of the oligonucleotides used in this study.

Name	Sequence (5′ → 3′)
MB	Biotin-TEG-6-FAMdT-CCGCGCTGTTAATGCTAATATGTAGGAGGCGCGG-TAMRA
mir-155-3p	CUCCUACAUAUUAGCAUUAACA
mir-155-5p	UUAAUGCUAAUCGUGAUAGGGGUU

## Data Availability

Data are contained within the article.
